# Oncogenic role of early growth response-1 in liver cancer through the regulation of the microRNA-675/sestrin 3 and the Wnt/β-catenin signaling pathway

**DOI:** 10.1080/21655979.2021.1964889

**Published:** 2021-08-19

**Authors:** Lingling Zhang, Ran Ren, Xue Yang, Yiman Ge, Xiajun Zhang, Hongping Yuan

**Affiliations:** a Department of Clinical Laboratory, Hospital of Chengdu University of Traditional Chinese Medicine, Chengdu, Sichuan, China; b Department of Clinical Laboratory, Danyang People’s Hospital, Zhenjiang, Jiangsu, China

**Keywords:** EGR1, miR-675, SESN3, Wnt/β-catenin, LC

## Abstract

Early growth response-1 (EGR1) is a multi-domain protein and an immediate early transcription factor that is induced during liver injury and controls the expression of a variety of genes implicated in metabolism, cell proliferation, and tumorigenesis. Liver cancer (LC) is a highly malignant disease with high mortality worldwide. This study focused on the function of EGR1 in LC development and the mechanism of action. Two LC-related datasets GSE101728 and GSE138178 downloaded from the Gene Expression Omnibus (GEO) database were used for identification of key genes involved in cancer progression. A microarray analysis was conducted to identify differentially expressed microRNAs (miRNAs) after EGR1 knockdown. The target gene of miR-675 was identified by integrated analysis. EGR1 and miR-675 were highly expressed, whereas sestrin 3 (SESN3) was poorly expressed in LC tissues and cells. High EGR1 expression was associated with poor liver function and disease severity in patients with LC. Knockdown of EGR1 weakened proliferation and invasiveness of LC cells. EGR1 bound to the miR-675 promoter and increased its transcription, and miR-675 bound to SESN3 mRNA to induce its downregulation. miR-675 upregulation promoted the malignance of LC cells, but further upregulation of SESN3 reduced invasiveness of cells. SESN3 was enriched in the Wnt/β-catenin signaling. EGR1 and miR-675 activated the Wnt/β-catenin through downregulating SESN3. This study demonstrated that EGR1 promotes the malignant behaviors of LC cells through mediating the miRNA-675/SESN3/Wnt/β-catenin axis.

## Introduction

Liver cancer (LC) is a highly malignant disease which ranks the 7^th^ most common cancer and 2^nd^ leading contributor to cancer-related deaths, with an estimated 840,000 diagnoses and 782,000 deaths annually in 2018 [1]. In 2021, liver cancer is estimated to rank the 11^th^ in terms of the number of new cases whereas rank the 5^th^ in terms of the number of deaths, and the incidence of LC is projected to continually rise in women by > 2% annually while stabilize in men [2]. Hepatocellular carcinoma (HCC) represents the predominant type of LC [3,4]. Surgical resection, ablation and liver transplantation are potential curative therapies for LC, though such options are only effective for early diagnosed patients without local or distant metastasis [5]. However, most patients are diagnosed at late stages, and the 5-year survival rate was no more than 5% due to the recurrence and metastasis [6]. In addition to an early diagnosis, identifying more molecular mechanisms implicated in the LC progression is of great significance for the development of potential therapeutic options.

Early growth response-1 (EGR1) is a multi-domain protein and a transcription factor that participates in multiple key physiological processes including development, metabolism and proliferation, and in cancer development [7]. The tumorigenic role of EGR-1 has been first revealed in prostate cancer, where high expression of EGR1 triggered cell proliferation and metastasis, leaving EGR1 as a candidate target for the therapy of prostate cancer [8,9]. As a transcription factor, EGR1 has been implicated in the carcinogenesis of breast cancer [10] and gastric cancer [11] and so forth through regulating the transcription activity of specific target genes. In the present study, high expression of EGR1 was found in LC according to the bioinformatic analyses using Gene Expression Omnibus (GEO, https://www.ncbi.nlm.nih.gov/geo/) datasets, and the microarray analysis suggested that microRNA (miRNA)-675 was significantly upregulated upon EGR1 overexpression.

miRNAs are a large class of non-coding RNAs that are usually deregulated during cancer progression and play versatile functions through binding to diverse target mRNAs [12]. miR-675 is an important derivative of the H19, a long non-coding RNA that is only expressed from the maternal allele and located in the telomeric region of chromosome 11p15.5 [13]. H19 is involved in embryonic and organ development and also related to the development of a multitude of human malignancies, during which the miR-675 is frequently involved [14]. Upregulation of H19 and miR-675 reduced cell apoptosis in hepatitis B virus-induced hepatoblastoma [15]. Therefore, it can be inferred that EGR1 might bind to miR-675 promoter to augment LC development. The subsequent integrated bioinformatics analyses predicted sestrin 3 (SESN3) as a target mRNA of miR-675. SESN3 is a member of a small unique protein family that do not share domain structures with any other eukaryotic proteins [16]. SESN proteins have been implicated in multiple biological functions such as protection against oxidative and genotoxic stresses, AMP-activated protein kinase and mechanistic target of rapamycin kinase signal transduction, and metabolic homeostasis [17]. SESN3 has been reported to protect against diet-induced nonalcoholic steatohepatitis in mice [17]. In addition, SESN3 deficiency has been demonstrated to promote HCC progression [18]. Taken together, we hypothesized that high expression of EGR1 in LC activates transcription of miR-675 and suppresses SESN3 expression, therefore triggering the pathological process of LC. Therefore, altered expression of EGR1, miR-675 and SESN3 was introduced in LC cells and both *in vitro* and *in vivo* experiments were conducted to validate their interactions and functions in LC development.

## Materials and methods

### Bioinformatics analyses

Two LC-related gene datasets GSE101728 and GSE138178 were acquired from the GEO database and analyzed using an Edge R/Bioconductor Package (NIH) of the R Program (Version 3.6.3, R). Differentially expressed genes (DEGs) were analyzed using |FoldChange| > 2 and *p* < 0.01 as the screening criteria. The heatmaps were produced using a heatmap package (version 3.6.3, R).

A Kyoto Encyclopedia of Genes and Genomes (KEGG) pathway analysis was conducted based on the DEGs and the predicted target genes of miR-675 using a ClusterProfiler/Bioconductor Package. The result was visualized using a Barplot Package (version 3.6.3, R). The target genes of miR-675 were predicted from the StarBase system (http://starbase.sysu.edu.cn/). The functional annotation and signaling information were obtained from https://www.kegg.jp/. The enrichment degree of signaling pathways was analyzed using Fishers’ exact test. The diagram of multivariate correlation analysis was produced using a Corregram Package (Version 3.6.3, R), and the correlations were analyzed by the Pearson’s correlation analysis. The promoter sequence of miR-675 was obtained from the UCSC website (https://genome.ucsc.edu/index.html). The binding site between miR-675 and SESN3 mRNA was predicted on ALEGEEN (http://alggen.lsi.upc.es/cgi-bin/promo_v3/promo).

### Clinical samples

Forty-three patients with primary LC treated in Hospital of Chengdu University of Traditional Chinese Medicine from September 2017 to September 2019 were included into this study. The patients were diagnosed by imaging examination and tissue biopsy, and the expected survival time of the included respondents was over 3 months. All included patients had complete clinical information. None of them received targeting therapies such as surgery, chemotherapy or radiotherapy. The patients who did not have a family companion, had deficiency in autoimmune system, got infected before admission, or refused to provide samples, were excluded. The peripheral blood, tumor tissues and the paracancerous tissues (> 5 cm away from the tumor sites) were harvested for further use. This study was ratified by the Ethical Committee of Hospital of Chengdu University of Traditional Chinese Medicine and in compliance with the *Declaration of Helsinki*. Written informed consent was collected from all subjects.

### Reverse transcription quantitative polymerase chain reaction (RT-qPCR)

RNA samples were collected using a TRIzol kit (Takara Holdings Inc., Kyoto, Japan). mRNA and miRNA were reverse-transcribed to complementary DNA (cDNA) using a RT kit (Thermo Fisher Scientific Inc., Waltham, MA, USA) and a miRNA First-Strand cDNA Synthesis Kit (TianGen Biotech Co., Ltd., Beijing, China), respectively. After that, real-time qPCR was conducted using a Premix Ex Taq DNA PCR kit (Takara) or a miRcute miRNA qPCR kit (TianGen Biotech), respectively, on an ABI Quant Studio 3 System (Applied Biosystems Inc., Foster City, CA, USA). The primer sequences are presented in Table 1, in which glyceraldehyde-3-phosphate dehydrogenase (GAPDH) and U6 were used as internal controls [19]. The 2^−ΔΔCt^ method was applied to quantify gene expression.

**Table 1.** Primer sequences for RT-qPCR

Gene	Primer sequence (5’-3’)
EGR1	F: CCTATGAGCACCTGACCACA
	R: ATCGTTTGGCTGGGATAACTC
miR-675	F: TGGTGCGGAGAGGGC
	R: GAACATGTCTGCGTATCTC
SESN3	F: CCGCCAGTAACTATCATACATGCG
	R: GAGGATGTTGACACAACCAT
GAPDH	F: CGGAGTCAACGGATTTGGTCGTAT
	R: AGCCTTCTCCATGGTGGTGAAGAC
U6	F: CGCAAGGATGACACGCAAAT
	R: ATTTGCGTGTCATCCTTGCG

Note: RT-qPCR, reverse transcription quantitative polymerase chain reaction; miR-675, microRNA-675; SESN3, sestrin 3; GAPDH, glyceraldehyde-3-phosphate dehydrogenase; F: forward; R, reverse

### Cell transfection

Four LC cell lines (Huh-7, HLE, Hep3B and MHCC97-H) and an immortalized human hepatocyte cell line THLE-3 were procured from ATCC (Manassas, VA, USA). The Huh-7 is a highly differentiated immortalized cell line composed of epithelioid tumorigenic cells. HLE and Hep3B are highly activated cell lines isolated from childhood HCC. MHCC97-H is a highly metastatic clonal cell line of MHCC97. THLE-3 is a normal liver epithelial cell line used for control. According to the instructions of the culture medium, THLE-3 cells were cultured in Roswell Park Memorial Institute-1640 (Thermo Fisher Scientific), whereas the LC cells were cultivated in Dulbecco’s modified Eagle’s medium (DMEM) (Thermo Fisher Scientific) which is suitable for the rapid growth and adherence of cancer cells. The media were added with 10% fetal bovine serum (FBS) and renewed every two days. Cells were passaged after the cell confluence reached 80%. One day before transfection, cells were sorted on culture plates at 1 × 10^6^/well, and the cell density was kept at approximately 50%. The pcDNA 3.3 vectors (Promega Corp., Madison, Wisconsin, USA) containing small interfering RNA (siRNA) and negative control (NC) of EGR1 (si-EGR1 and EGR1-NC), containing miR-675 and control, or containing SESN3 and NC-SESN3 were transfected into cells according to the instructions of the Lipofectamine 3000 kit (Thermo Fisher scientific). In short, 2 μg recombinant vector was mixed in 375 μL serum-free medium, and 12 μL Lipofectamine 3000 was mixed in 375 μL serum-free medium as well. The two mixtures were incubated for 30 min and loaded into the cell plates. After incubation at 37°C with 5%CO_2_ for 6 h, the medium was refreshed. Cells were harvested for further experiments 48 h after transfection.

### Determination of biochemical factors

Venous blood samples from LC patients were collected and centrifuged at 3,000 g for 5 min. The concentrations of total bilirubin (TBIL), aspartate aminotransferase (AST) and alanine aminotransferase (ALT) in serum samples were examined using a Vitros350 analyzer (Johnson & Johnson, Chesterbrook, Pennsylvania, USA) and the potentiometric analysis for 5 min with a coefficient of variation of 1.13%-2.04%. The alpha-fetoprotein (AFP) concentration in serum was evaluated using an ADVIA Centaur XP Automatic chemiluminescence instrument (Siemens Ltd., Erlangen, Germany). The acridinium ester was used as the luminescent substrate, and the double antibody sandwich method was used.

### Colony formation assay

Proliferation of cells was examined using the colony formation assay. After transfection, the cells were trypsinized and resuspended in phosphate-buffered saline (PBS). Then, 5 × 10^3^ cells were cultured in 6-well plates at 37°C with 5% CO_2_ for 10 d till the appearance of visible cell colonies. The colonies were fixed in 4% formaldehyde (5 mL) and then stained with crystal violet for 20 min. The formation of colonies (over 10 cells) was captured under a DM4000B microscope (Leica, Solms, Germany).

### Transwell assay

Transwell assays were performed to examine the migration and invasion activities of cancer cells [20]. Transwell chambers (Millipore Corp., Billerica, MA, USA) were inserted into 24-well plates. Each apical chamber was pre-coated with 30 μL Matrigel (Becton-Dickinson (BD) Bioscience, San Jose, CA, USA). Transfected HLE and Hep3B cells were incubated in 300 μL serum-free DMEM in apical chambers at 8 × 10^4^ cells per well. Each basolateral chamber was loaded with 600 μL 10% FBS-contained DMEM. The cells were cultured at 37°C for 72 h. Then, cells invaded to the lower membranes were fixed and stained with crystal violet. Migration of cells was determined in a similar manner except for pre-coating Matrigel on the apical chambers. The number of migrating and invading cells was counted under the microscope.

### Cell apoptosis detection

Cell apoptosis was evaluated using Hoechst 33,258 staining (Beyotime Biotechnology Co., Ltd., Shanghai, China). Cell samples were collected and loaded in a 1.5-mL centrifuge tube to discard the supernatant. Then, cells were fixed in 0.5 mL fixing solution for 10 min and loaded on glass slides. After adherence, the cells were stained with Hoechst 33,258 staining solution (0.5 mL) for 5 min. The cell slides were further loaded with anti-fluorescence quenching solution. The staining was observed under a fluorescence microscope at an excitation wavelength of 350 nm and an emission wavelength of 460 nm. The nuclei of apoptotic cells were stained in blue under the microscope [21].

Flow cytometry was further performed to examine the apoptosis rate in LC cells. After transfection, exponentially growing cells were washed in cold PBS and resuspended in binding buffer. The apoptotic cells were stained with Annexin V-fluorescein isothiocyanate/propidium iodide at room temperature in the dark for 15 min using an Annexin-V-FLUOS staining kit (Roche Ltd, Basel, Switzerland). The fluorescence signals were collected by FACSCanto (BD Bioscience, San Jose, CA, USA) and analyzed by the FlowJo 8.7.1 software (Ashland, OR, USA) [22].

### Tumor formation in nude mice

Xenograft tumors were induced in mice to examine the tumorigenic activity of cells *in vivo* [23]. Thirty-six BALB/c nude mice (20 ± 2 g, 4–5 weeks old) were provided by the Vital River Laboratory Animal Technology Co., Ltd. (Beijing, China). All animals were cultured in a 12-h dark/light cycle at 25 ± 2°C with 50～60% humidity. They were allowed to get free access to standard rodent feed and water. The usage of animals was approved by the Animal Ethics Committee of Hospital of Chengdu University of Traditional Chinese Medicine. All procedures were carried out in compliance with the Guidelines for animal care and use (NIH, Bethesda, MA, USA). The mice were randomly allocated into 6 groups including si-EGR1, EGR1-NC, si-EGR1 + miR-675, si-EGR1 + control, miR-675 + SESN3, and miR-675 + NC groups. Each mouse was transplanted with 8.0 × 10^6^ HLE or Hep3B cells (resuspended in 100 mL PBS) with stable transfection of pcDNA vectors. Then, the length (L) and width (W) of the xenograft tumors were examined every 7 d using a vernier caliper, and the tumor volume (V) was determined as follows: V = (L × W/2)^3^ × 0.5236. On the 28^th^ d, the animals were sacrificed via injection of 150 mg/kg 1% pentobarbital sodium (intraperitoneal injection) to collect the tumors. The tumors were weighed and used for histological staining.

### Immunohistochemical staining

Immunohistochemical staining was performed to examine the expression of the proliferation marker Ki-67 in xenograft tumors. The xenograft tumors from mice were prepared as 4-μm sections and dewaxed and rehydrated. After antigen retrieval in water bath for 1 h, the sections were blocked with normal goat serum (Solarbio Science & Technology Co., Ltd., Beijing, China) at 22°C for 20 min. After that, the sections were hybridized with anti-Ki-67 (1:100, ab15580. Abcam Inc., Cambridge, MA, USA) at 4°C for 15 h, and then with goat anti-rabbit immunoglobulin G (IgG, 1:100, ab7817, Abcam) and horseradish peroxidase (HRP)-labeled streptavidin (Solarbio) at 37°C for 20 min. The tissue sections were further treated with 3,3ʹ-diaminobenzidine for color development and then counter-stained with hematoxylin (Solarbio) for 1 min. After that, the tissues were dehydrated, cleared in xylene and sealed by neutral resin, and observed under the microscope.

### Metastasis of xenograft tumors in mice

Another 12 BALB/c mice were utilized for metastasis assay to examine cell metastasis activity *in vivo* [21]. In short, 1 × 10^6^ cells with stable transfection were resuspended in 100 mL PBS and transplanted into mice through caudal vein injection. After 48 d, the mice were euthanized and the lung tissues were collected for hematoxylin and eosin (HE) staining.

### HE staining

HE staining was performed to examine the number of metastatic nodules in mouse lung tissues. The murine lung tissues were fixed in Bouin’s fixative for 6 h, dehydrated in alcohol, prepared as 5-μm sections and rehydrated. A HE staining kit (Beyotime) was used. The tissue sections were soaked in hematoxylin solution for 12 min, in hydrochloric acid-ethanol for 10 s, and then in eosin solution for 4 min. After staining, the sections were dehydrated, cleared, sealed, and observed under the microscope.

### Microarray analysis

The microarray analysis was conducted to identify differentially expressed miRNAs after EGR1 treatment. The TRIzol Reagent was used to extract total RNA. The RNA (100 ng) was dephosphorylated by calf intestinal alkaline phosphatase (GE Healthcare Europe GmbH, Penzberg, Germany) and denaturalized in dimethyl sulfoxide. The RNA was further labeled with pCp-Cy3 using a T4 RNA ligase (GE Healthcare). The labeled RNA was hybridized with Aglient miRNA microarray (Agilent Technologies, Palo Alto, CA, USA) for 20 h. Then, the microarrays were scanned using an Agilent SureScan Microarray scanner (Agilent). The data were extracted using an Agilent feature extraction 10.0 (Aglient) and the corresponding heatmaps were produced.

### Chromatin immunoprecipitation (ChIP)-qPCR

The ChIP assay was conducted to examine the enrichment of EGR1 on miR-675. When an 80% cell confluence was reached, the Hep3B cells were analyzed using a Simple ChIP Enzymatic Chromatin IP Kit (Cell Signaling Technology, Beverly, MA, USA). The cells were cultured in culture dishes supplemented with 37% formaldehyde at 22°C for 10 min. The chromatin was obtained by ultrasonic treatment, and the chromatin samples were loaded with nuclease-free water, NaCl and RNAse A, and further incubated with 2 μL Proteinase K at 65°C for 2 h. The DNA was diluted in ChIP Buffer and then hybridized with Protein G Magnetic Beads at 4°C for 2 h. Next, the chromatin was eluted from the antibody/Protein G Magnetic Beads and crosslinked. The DNA was purified using spin columns. The DNA concentration in immunoprecipitates was evaluated by qPCR.

### Luciferase assay

The dual luciferase reporter gene assay was performed to examine the binding relationship between miR-675 and SESN3 mRNA [24]. The SESN3 3ʹuntranslated region (3ʹUTR) fragment (wild-type, WT) containing the binding site with miR-675 was amplified and sub-cloned to the downstream of the pmirGLO vectors (Promega), which was named SESN3-WT. The mutant-type (MT) vector was established using the mutant binding sequences and named SESN3-MT. The SESN3-WT/SESN3-MT vectors were transfected with miR-675 inhibitor or the inhibitor control into HEK293T cells. After 24 h, the luciferase activity in cells was examined according to the protocol of a Dual-Luciferase® Reporter Assay System (E1910, Promega).

### Western blot assay

Western blot analysis was conducted to detect the protein levels and signaling pathway activity in cells [25]. Total protein in cells was collected in radio-immunoprecipitation assay lysis buffer (Beyotime) to collect. A Protein Quantification Kit-Rapid (Sigma-Aldrich Chemical Company, St Louis, MO, USA) was utilized to examine the protein concentration. Next, the protein sample was separated on sodium dodecyl sulfate-polyacrylamide gel electrophoresis and loaded on polyvinylidene fluoride membranes (Bio-Rad Laboratories, Hercules, CA, USA). Next, the membranes were blocked for 2 h in nonfat milk and hybridized with the primary antibodies against β-catenin (sc-7963,1:500, Santa Cruz Biotechnology Inc., Santa Cruz, CA, USA), c-myc (sc-40, 1:2,000, Santa Cruz) and GAPDH (ab8245, 1:2,000, Abcam) at 4°C overnight. Next, the membranes were incubated with the HRP-labeled IgG (ab205719, 1:5,000, Abcam) at 37°C for 1 h. The protein bands were visualized using the enhanced chemiluminescence reagent. The images were collected using the Quantity One Software (Bio-Rad).

### Statistical analysis

Data analysis was performed using Statistical Product and Service Solutions 22.0 (IBM Corp. Armonk, NY, USA). Measurement data were collected from three repetitions and presented as the mean ± standard deviation (SD). Differences were analyzed by the *t* test (two groups) or one-way or two-way analysis of variance (ANOVA, multiple groups). Correlation between different factors was determined by Pearson’s correlation analysis. The receiver operating characteristic (ROC) curve was produced and the area under curve (AUC) was analyzed by Wilson/Brown analysis. *p < 0.05 was considered to show statistically significant difference.

## Results

### EGR1 is highly expressed in LC samples

**Figure 1.**
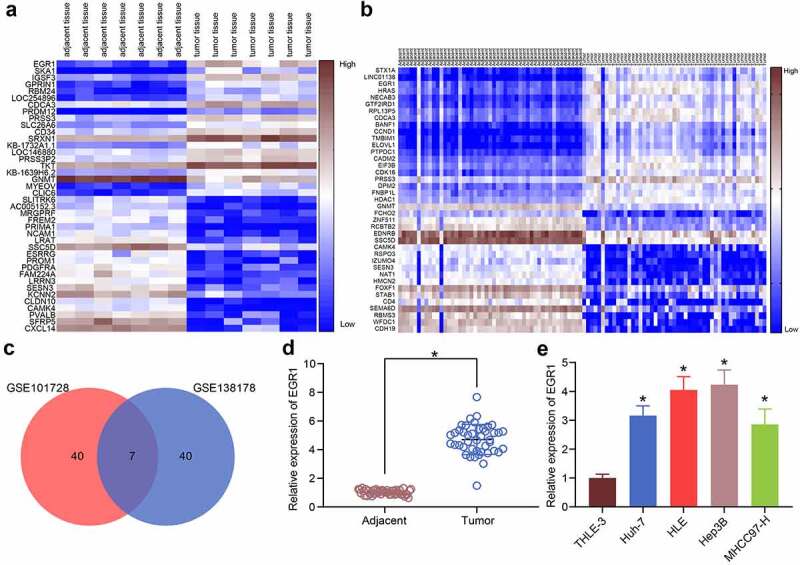
EGR1 is highly expressed in LC samples. a-b, heatmaps for DEGs between LC tissues and healthy tissues in the GEO GSE101728 (a) and GSE138178 (b) datasets; c, an intersection analysis based on the findings in a and b using a Venn diagram; d, expression of EGR1 mRNA in the clinical tumor tissues and the para-cancerous tissues examined by RT-qPCR (n = 43, the paired *t* test, *p < 0.05); e, expression of EGR1 mRNA in the acquired LC cell lines (Huh-7, HLE, Hep3B and MHCC97-H) and in THLE-3 cells evaluated by RT-qPCR (one-way ANOVA, *p < 0.05)

Aberrantly expressed mRNAs in LC were predicted using GEO datasets. First, the GSE101728 dataset containing 7 pairs of LC tissues and para-cancerous tissues and another GSE138178 dataset containing cancer and para-cancerous tissue from 49 patients were analyzed. The heatmaps were shown in Figure 1(a-b). The common outcomes of the two heatmaps were compared using a Venn diagram (Figure 1c), and a KEGG functional annotation was performed based on the DEGs (Table 2). EGR1 was found as a differentially expressed transcription factor in LC tissues. This attracted our attention to explore whether EGR1 affects pathological process of LC and the mechanism of action. Thereafter, the RT-qPCR results suggested that the expression of EGR1 was elevated in the clinical tumor tissues versus that in the para-cancerous tissues (Figure 1d). Likewise, high expression of EGR1 was observed in the acquired LC cell lines Huh-7, HLE, Hep3B and MHCC97-H versus that in THLE-3 cells (Figure 1e).

**Table 2.** A KEGG functional annotation for the differentially expressed genes from GSE101728 and GSE138178

Gene name	KEGG ID	Functional annotation
CDCA3	83461	Cell division cycle associated 3
EGR1	1958	Early growth response protein 1
PRSS3	167681	Serine protease 35
SESN3	143686	Sestrin 1/3
GNMT	27232	Glycine N-methyltransferase
SSC5D	284297	Scavenger receptor cysteine rich family member with 5 domains
CAMK4	461992	Calcium/calmodulin-dependent protein kinase IV

Note: KEGG, Kyoto Encyclopedia of Genes and Genomes;

### EGR1 serves as a potential biomarker of LC

**Figure 2.**
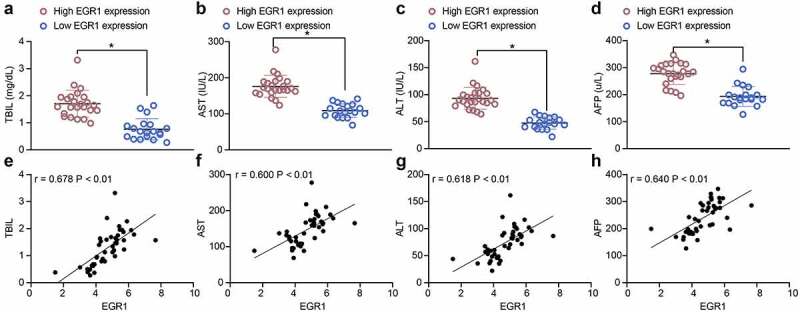
EGR1 serves as a potential biomarker of LC. a-s, levels of liver function-related factors TBIL (a), AST (b) and ALT (c) in the serum of LC patients (median expression of EGR1 = 4.24, *p < 0.05, unpaired *t* test); d, level of the LC-specific marker protein AFP in the serum of LC patients (median expression of EGR1 = 4.24, *p < 0.05, unpaired *t* test); e-h, positive correlations between EGR1 and the expression of TBIL (e), AST (f), ALT (g) and AFP (h) (Pearson’s correlation analysis, all *p < 0.05)

To explore the correlation between EGR1 and the clinical parameters of LC patients, the baseline characteristics of the included patients were analyzed (Table 3). It was found the EGR1 expression was not relevant to the age and sex of patients but correlated with advanced tumor node metastasis (TNM) staging and increased tumor size. Next, the correlations between EGR1 expression and the serum levels of liver impairment-related factors including TBIL (Figure 2a), AST (Figure 2b) and ALT (Figure 2c) in patients were analyzed. All these factors were upregulated in patients owning high expression of EGR1. In addition, the expression of the LC marker protein AFP in the serum of patients was examined as well. Likewise, a significant increase in AFP expression was found in patients with high expression of EGR1 (Figure 2d). The subsequent Pearson’s analysis suggested that the EGR1 expression was positively correlated with the expression of TBIL, AST, ALT and AFP in patients (Figure 2(e-h)). These results indicated that EGR1 may serve as a biomarker indicating dismal prognosis in LC patients.

**Table 3.** Correlation between EGR1 expression with the clinical baseline characteristics of patients with LC

Clinical characteristics		EGR1		*p*
		High	Low	
Age	≤ 65	11	9	> 0.05
	> 65	13	10	
Sex	Female	14	12	> 0.05
	Male	10	7	
Size (cm)	≤ 4	6	10	< 0.05
	> 4	18	9	
TNM stage	I-II	7	13	< 0.05
	III-IV	17	6	
TBIL (mg/dL)		1.56 ± 0.36	0.62 ± 0.25	< 0.05
AST (IU/L)		185.69 ± 32.17	102.75 ± 23.74	< 0.05
ALT (IU/L)		95.71 ± 16.72	47.56 ± 7.42	< 0.05
AFP (U/L)		281.52 ± 62.15	192.71 ± 31.57	< 0.05

Note: EGR1, early growth response 1; LC, liver cancer; TNM, tumor node metastasis; TBIL, total bilirubin; AST, aspartate aminotransferase; ALT, alanine aminotransferase; AFP, alpha-fetoprotein

### Artificial downregulation of EGR1 reduces viability of LC cells

**Figure 3.**
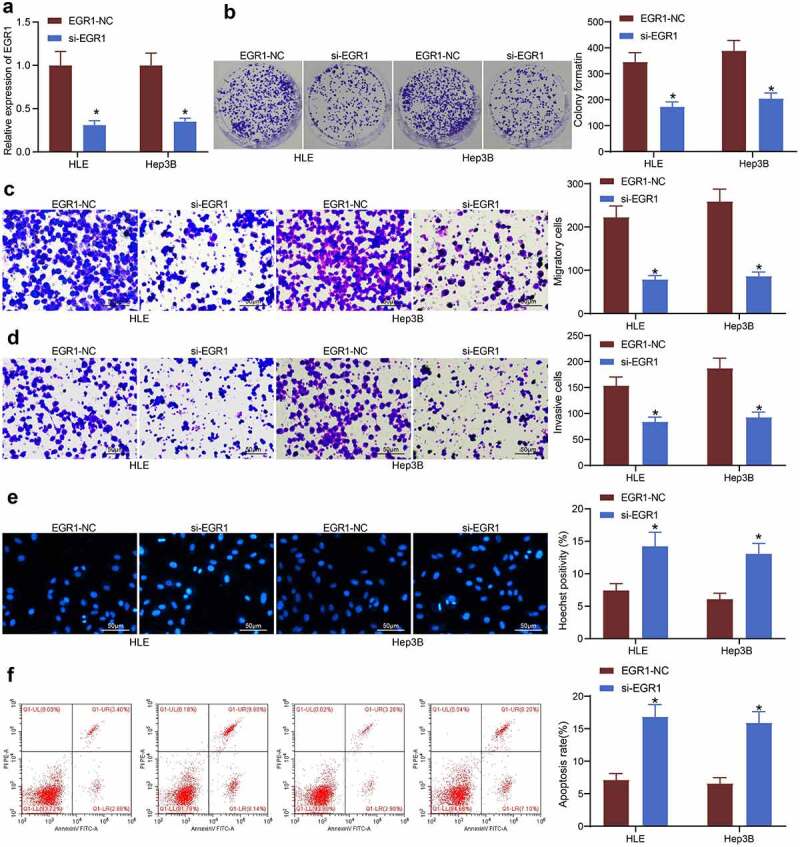
Artificial downregulation of EGR1 reduces viability of LC cells. a, EGR1 expression in HLE and Hep3B cells detected by RT-qPCR (two-way ANOVA, *p < 0.05); b, proliferation of HLE and Hep3B cells determined by colony formation assays (two-way ANOVA, *p < 0.05); c-d, migratory (c) and invasive (d) potentials of HLE and Hep3B cells determined by Transwell assays (two-way ANOVA, *p < 0.05); e, apoptosis of HLE and Hep3B cells evaluated by Hoechst 33,258 staining (two-way ANOVA, *p < 0.05); F, apoptosis rate in HLE and Hep3B cells evaluated by flow cytometry (two-way ANOVA, *p < 0.05)

To explore the exact functions of EGR1 in the behaviors of LC cells, artificial suppression of EGR1 was induced in HLE and Hep3B cells via transfection of si-EGR1 (Figure 3a). Thereafter, the viability of HLE and Hep3B cells was first determined through a colony formation assay. It was found that the formation of cell colonies in given time was declined upon EGR1 downregulation (Figure 3b). In addition, the Transwell assays showed that the migration (Figure 3c) and invasion (Figure 3d) of cells were decreased when EGR1 was suppressed. The apoptosis of cells, according to the Hoechst 33,258 staining, was significantly increased after EGR1 downregulation (Figure 3e). In agreement with this, the subsequent flow cytometry results also indicated that the apoptosis activity in cells was increased upon EGR1 silencing (Figure 3f).

### Downregulation of EGR1 reduces LC tumorigenesis in nude mice

**Figure 4.**
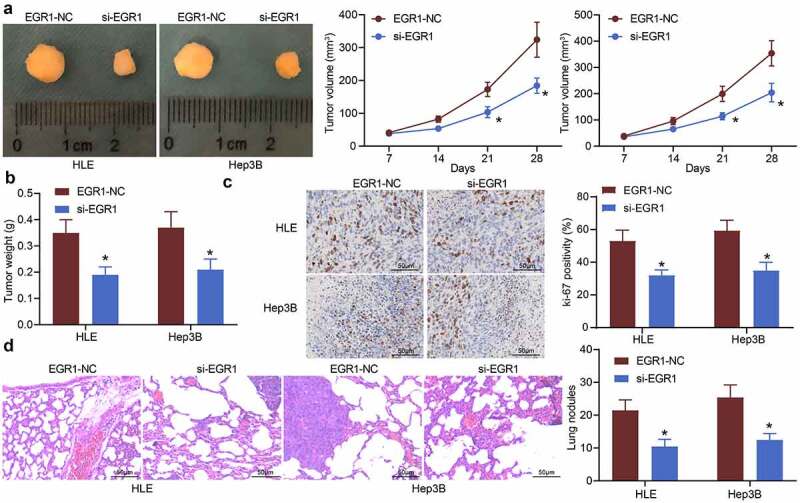
Downregulation of EGR1 reduces LC tumorigenesis in nude mice. a, volume of the xenograft tumors in nude mice (two-way ANOVA, *p < 0.05); b, weight of tumors on the 28^th^ d (two-way ANOVA, *p < 0.05); c, expression of in the tumor tissues detected by immunohistochemical staining (two-way ANOVA, *p < 0.05); d, formation of metastatic nodules in murine lung tissues assessed by HE staining (two-way ANOVA, *p < 0.05)

*In vivo* experiments were performed by transplanting HLE and Hep3B cells into nude mice via subcutaneous injection. The tumor volume in mice was examined. Since the 3^rd^ week, the volume of tumors formed by cells transfected with si-EGR1 was significantly reduced (Figure 4a). The mice were sacrificed on the 28^th^ d, and the tumor tissues were weighed. As expected, the weight of tumors in mice was reduced as well upon EGR1 downregulation (Figure 4b). Next, the tissues were harvested for immunohistochemical staining. It was found that the positive staining of the proliferation marker Ki-67 in tumor tissues was reduced after EGR1 suppression (Figure 4c). The metastasis of xenograft tumors was evaluated by transplanting HLE and Hep3B cells into mice through caudal veins. On the 48^th^ d, the mice were sacrificed and the lung tissues were harvested. The subsequent HE staining showed that the formation of metastatic nodules in lung tissues was significantly declined after EGR1 silencing (Figure 4d).

### EGR1 transcriptionally activates miR-675 to suppress SESN3 expression

**Figure 5.**
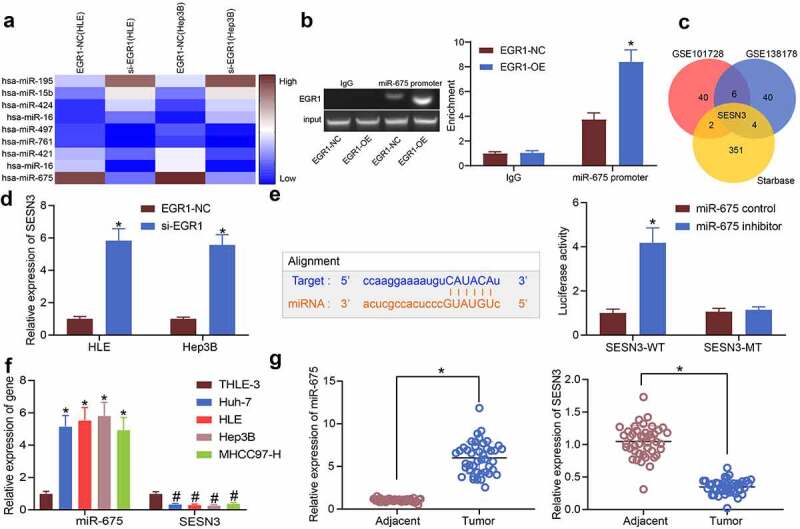
EGR1 transcriptionally activates miR-675 to govern SESN3 expression. a, differentially expressed miRNAs in cells transfected with si-EGR1 determined using a miRNA microarray analysis; b, binding between EGR1 and miR-675 promoter validated via the ChIP-qPCR assay (two-way ANOVA, *p < 0.05); c, a Venn map for the intersection of the predicted mRNA targets of miR-675 and the differentially expressed mRNAs of the two GEO datasets; d, mRNA expression of SESN3 in HLE and Hep3B cells after si-EGR1 transfection examined by RT-qPCR (two-way ANOVA, *p < 0.05); e, binding between miR-675 and SESN3 validated via a luciferase assay (two-way ANOVA, *p < 0.05); f, miR-675 and SESN3 expression in the LC cell lines (Huh-7, HLE, Hep3B and MHCC97-H) and in THLE-3 cells quantified by RT-qPCR (two-way ANOVA, *#*p* < 0.05); g, expression of miR-675 and SESN3 mRNA in the LC tissues and the para-cancerous tissues examined by RT-qPCR (n = 43, the paired t test)

To further investigate the potential molecules of action, we first explored the differentially expressed miRNAs in cells transfected with si-EGR1 using a miRNA microarray analysis, which suggested that miR-675 was downregulated in cells in a highest degree with the decline of EGR1 (Figure 5a). Next, the putative binding sequence between EGR1 and the miR-675 promoter was predicted on ALGGEN and validated through a ChIP assay. Overexpression of EGR1 was introduced in Hep3B cells, and an enrichment of EGR1 protein was found on miR-675 promoter in the immunoprecipitates (Figure 5b), indicating that EGR1 can bind to miR-675 promoter and regulate miR-675 transcription. We next predicted the target genes of miR-675 on the bioinformatics system StarBase, and the outcomes were compared with the DEGs screened by two GEO datasets above. Importantly, SESN3 was analyzed as a target of miR-675 that was poorly expressed in LC tissues (Figure 5c). Next, the RT-qPCR results indicated that the SESN3 expression was significantly increased in HLE and Hep3B cells transfected with si-EGR1 (Figure 5d). The binding between miR-675 and SESN3 3ʹUTR was further verified using a luciferase assay (Figure 5e). It was suggested that miR-675 inhibitor increased luciferase activity of the SESN3-WT vector in cells. The expression of miR-675 and SESN3 mRNA in the acquired cell lines was determined. Importantly, miR-675 was significantly highly expressed whereas SESN3 was poorly expressed in the LC cells, particularly in HLE and Hep3B cells, versus the THLE-3 cells (Figure 5f). Similar trends were observed in the collected LC tumors and the paired adjacent ones (Figure 5g). Therefore, we speculated that EGR1 transcriptionally activates miR-675 to suppress SESN3 expression.

### miR-675 and SESN3 are potential biomarkers of LC

**Figure 6.**
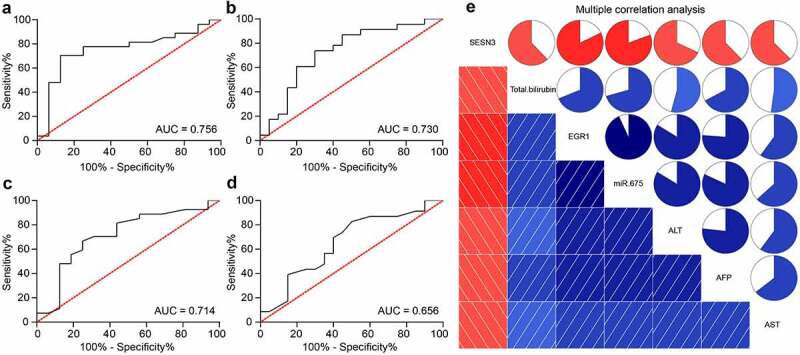
miR-675 and SESN3 are possible biomarkers of LC. a, predictive value of miR-675 in tumor size (a) and TNM staining (b) determined by ROCs (Wilson/Brown analysis, *p< 0.05); c-d, predictive value of SESN3 in tumor size (c) and TNM staining (d) determined by ROC (Wilson/Brown analysis, *p< 0.05); e, interactions among EGR1, miR-675 and SESN3 expression and the levels of TBIL, AST, ALT and AFP in serum of patients analyzed using a multivariate correlation analysis (Pearson’s analysis, *p< 0.05)

The findings above suggested that EGR1 was positively associated with the tumor size, TNM staging, and the serum expression of TBIL, AST, ALT and AFP in patients. We further explored whether miR-675 and SESN3 can serve as biomarkers of LC. TheROC was produced to analyze the relevance of miR-675 to the characteristics of LC in patients. It was found that miR-675 could effectively predict the tumor size, and the AUC was 0.756 (Figure 6a). Also, the ROC suggested that miR-675 expression was specifically linked to TNM staging, and the AUC was 0.730 (Figure 6b). The correlations between SESN3 expression and the tumor size and TNM staging were analyzed as well. The AUC was 0.714 and 0.656, respectively (Figure 6(c-d)). In addition, a multivariate correlation analysis was conducted to explore the correlations between the EGR1, miR-675 and SESN3 expression and the serum levels of TBIL, AST, ALT and AFP in patients. In the diagram below (Figure 6e), the EGR1 and miR-675 were positively correlated with the levels of TBIL, AST, ALT and AFP in the serum of patients (blue), but SESN3 expression was negatively associated with the levels of the serum TBIL, AST, ALT and AFP levels (red). Although whether the impact of EGR1/miR-675/SESN3 on the TBIL, AST, ALT and AFP levels is direct or not was unknown, it could be inferred that that there are specific correlations between EGR1/miR-675/SESN3 and the TBIL, AST, ALT and AFP levels. Also, EGR1 was positively correlated with miR-675 expression, but SESN3 was negatively correlated with the expression of EGR1 and miR-675. According to the correlation degree (reflected by the color proportion in the upper right diagrams), EGR1 showed a particularly high correlation with miR-675, and the correlations between other variables were also significant and exceeded 50%. Collectively, miR-675 and SESN3 may also serve as biomarkers of LC.

### miR-675 and SESN3 affects viability and aggressiveness of LC cells

**Figure 7.**
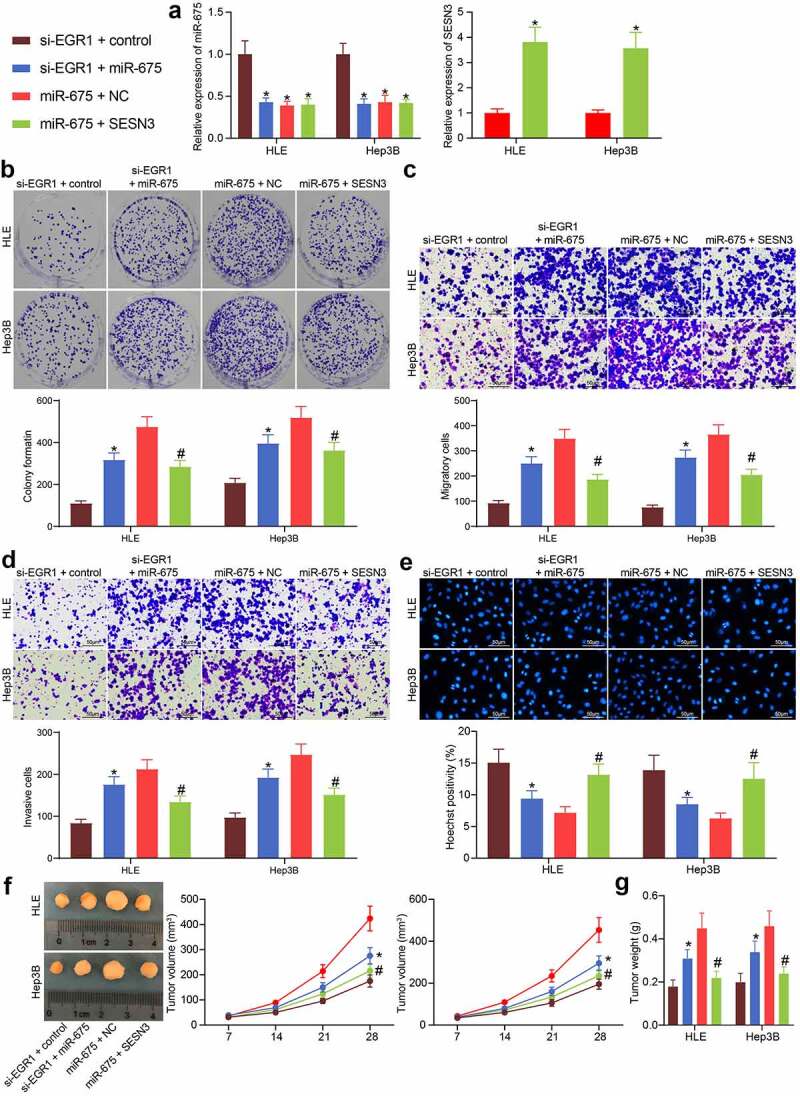
miR-675 and SESN3 affects viability and aggressiveness of LC cells. a, expression of miR-675 and SESN3 mRNA in HLE and Hep3B cells evaluated by RT-qPCR (two-way ANOVA, *p < 0.05); b, proliferation of HLE and Hep3B cells evaluated by colony formation assays (two-way ANOVA, *p < 0.05); c-d, migratory (c) and invasive (d) potentials of HLE and Hep3B cells evaluated by Transwell assays (two-way ANOVA, *p < 0.05); e, apoptosis of HLE and Hep3B cells evaluated by Hoechst 33,258 staining (two-way ANOVA, *p < 0.05); f, volume of the tumors in nude mice (two-way ANOVA, *p < 0.05); g, weight of the tumors on the 28^th^ d (two-way ANOVA, *p < 0.05)

To further evaluate the functions of miR-675 and SESN3 in the activity of LC cells, the HLE and Hep-3B cells transfected with si-EGR1 were additionally transfected with miR-675, which were further transfected with SESN3 (Figure 7a). Then, it was found that the number of cell colonies was increased after miR-675 upregulation but then suppressed after SESN3 overexpression (Figure 7b). The Transwell assay results suggested that the migratory and invasive potentials of cells suppressed by si-EGR1 were recovered by miR-675 but then blocked again following SESN3 upregulation (Figure 7(c-d)). The Hoechst 33,258 staining showed that the cell apoptosis was reduced by miR-675 but increased by SESN3 (Figure 7e). Further, the *in vivo* experiments showed that the volume and weight of xenograft tumors in nude mice initially suppressed by si-EGR1 were increased after miR-675 upregulation. The additional overexpression of SESN3, still, blocked the growth of xenograft tumors induced by LC cells in mice (Figure 7(f-g)). These results validated the speculation above that EGR1 activates miR-675 and suppresses SESN3 expression to promote LC growth.

### The EGR1/miR-675/SESN3 axis mediates the Wnt/β-catenin pathway

**Figure 8.**
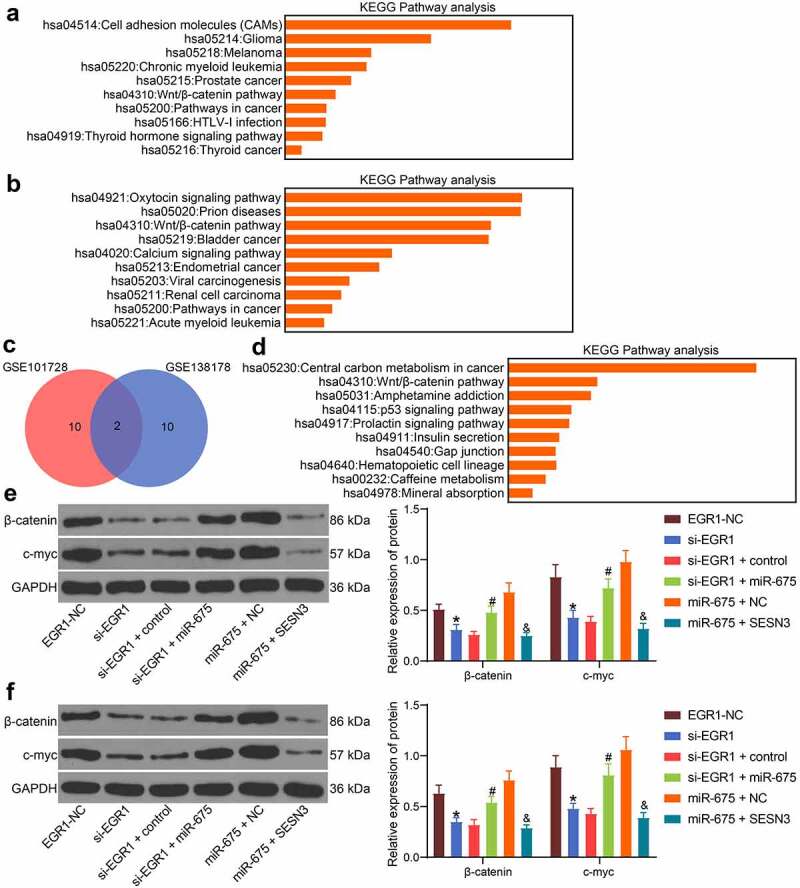
The EGR1/miR-675/SESN3 axis mediates the Wnt/β-catenin pathway. a-b, abnormally activated signaling pathways in LC analyzed by KEGG pathway analyses based on the genes screened using the GSE101728 (a) and GSE138178 (b) datasets, and the top 10 pathways were presented (* *p* < 0.05, Fisher’s exact test); c, a Venn map for the intersected pathways in panels a and b; d, a KEGG enrichment analysis based on the targeting genes of miR-675, and the top 10 pathways were presented (Fisher’s exact test, *p < 0.05); e-f, protein levels of β-catenin and c-myc in HLE (e) and Hep3B (f) cells examined by western blot analysis (two-way ANOVA, *p < 0.05 vs. EGR1-NC, #*p* < 0.05 vs. si-EGR1 + miR-675, &*p* < 0.05 vs miR-675+ SESN3)

KEGG pathway analyses were performed as well based on the DEGs screened by the GSE101728 and GSE138178 datasets (Figure 8(a-b)). The analysis results based on the two datasets confirmed a common enrichment of the Wnt/β-catenin pathway (Figure 8c). Coincidentally, EGR1 was predicted as an upstream regulator of this signaling pathway. Of note, another KEGG pathway enrichment analysis based on the predicted target genes of miR-675 suggested that many miR-675-targeted genes were enriched in the Wnt/β-catenin pathway as well, including SESN3 (Figure 8d). We therefore wondered that the EGR1/miR-675/SESN3 possibly regulates the Wnt/β-catenin pathway. Thereafter, the protein levels of β-catenin and c-myc in HLE cells were examined by western blot analysis. The activity of the Wnt/β-catenin pathway was suppressed by si-EGR1 but recovered after additional upregulation of miR-675. Still, further overexpression of SESN3 reduced the protein levels of β-catenin and c-myc in cells (Figure 8e). Similar results were reproduced in Hep3B cells that silencing of EGR1 or upregulation of SESN3 reduced whereas upregulation of miR-675 restored the protein levels of β-catenin and c-myc (Figure 8f).

## Discussion

Although most of the risk factors including smoking, alcohol consumption, obesity, and hepatitis B and C virus (HBV and HCV) infections are preventable, the occurrence of LC has risen most rapidly among all cancers by an annual 2% to 3% rise in the past decades [26]. Management of LC remains a huge challenge because of the high malignancy and aggressiveness of this disease. Researchers in this field have focused on identifying novel molecules responsible for LC development. Here, our study confirmed an oncogenic role of EGR1 in LC through a novel miR-675/SESN3/Wnt/β-catenin.

The GEO datasets comprising gene expression data generated by DNA microarray technology are advantage tools and helpful in screening gene expression profiles in different pathological conditions [27]. Here, data from GSE101728 and GSE138178 datasets suggested EGR1 as a notably upregulated transcription factor in LC samples. The following clinical analyses suggested that high EGR1 expression in patients was associated with increased tumor size and advanced TNM staging, and decreased liver function (reflected by increased expression of TBIL, AST and ALT) and LC severity (increased expression of AFP). The tumorigenic role of EGR1 was first revealed in prostate cancer where Egr1-deficient mice showed impaired tumorigenesis rate [28]. Also, EGR1 level was found to be relevant to the genes encoding angiogenic/osteoclastogenic pathway effectors and to directly affect cancer metastasis [9]. Poor expression of EGR1 increased the survival rate of patients with glioma, and EGR1 silencing suppressed proliferation but promoted cell cycle arrest of glioma cells [29]. More relevantly, high EGR1 expression was found in the tissues collected from HCC patients, and it encouraged transforming growth factor-β1-induced proliferation [24]. In our subsequent experiments, we found that artificial downregulation of EGR1 suppressed proliferation and metastasis but increased apoptosis of HLE and Hep3B cells. Similar trends were reproduced *in vivo* where EGR1 silencing reduced the growth and dissemination of tumors in mice. These results confirmed an oncogenic role of EGR1 in LC.

EGR1 has been demonstrated to inhibit the transcription activity of anti-oncogenic miR-195 and reduce the apoptosis of gastric cancer cells [11]. Increased nuclear translocation of EGR1 was found to enhance the transcription activity of miR-3928 v and downregulated expression of voltage-dependent anion channel 3, a tumor-suppressor, thereby increasing malignancy of LC cells [30]. We therefore explored the differentially expressed miRNAs in LC cells after EGR1 knockdown in our experiments, and miR-675 was confirmed as a target. Thereafter, we validated the binding between EGR1 and the miR-675 promoter and confirmed that EGR1 promoted miR-675 transcription. This was in concert with a previous report which suggested that HMGA1 Pseudogene 7 upregulates miR-675 and miR-483 through activating EGR1 [31]. Interestingly, miR-675 has been demonstrated to inhibit heterochromatin1 isoform HP1 alpha to reduce total histone H3 lysine 9 trimethylation and histone H3 lysine 27 trimethylation whereas increase histone H3 lysine 27 acetylation in EGR1 promoter, which consequently increased EGR1 transcription, translation and activation in LC and the subsequent H19 activation [32]. Therefore, there might be a positive feedback loop between EGR1 and miR-675 in LC. Furthermore, the target mRNAs of miR-675 were predicted on StarBase, and the outcomes were compared with the DEGs screened out using above GEO datasets, and SESN3 was identified to be intersected. The binding relationship between miR-675 and SESN3 mRNA was confirmed by the dual luciferase reporter gene assay. We next confirmed high miR-675 expression while poor SESN3 expression in the collected LC tissues and the acquired LC cell lines. miR-675 as well as its host gene H19 has been frequently observed as a cancer driver in many human malignancies such as gastric cancer [33], thyroid carcinoma [34,35], breast cancer [36] and nasalpharyngeal cancer [37]. In hepatoblastoma, miR-675-mediated downregulation of FADD was found to reduce cell apoptosis and promote tumorigenesis [15]. In addition, high expression of miR-675 was found in AFP-secreting HCC, which usually links to a significant rise in proliferative and growth capacity [38]. On the contrary, SESN3 has been suggested as a tumor-suppressor in HCC, since SESN3 deficiency promoted carcinogen-induced HCC via regulating the hedgehog pathway [39]. To validate the involvements of miR-675 and SESN3 in EGR1-mediated events. Further overexpression of miR-675 and SESN3 was introduced in LC cells pre-treated with si-EGR1, after which the inhibitory functions of si-EGR1 on LC cell growth were blocked after miR-675 overexpression, while overexpression of SESN3 reduced the growth and metastasis of LC cells again.

Importantly, our subsequent KEGG pathway enrichment analysis suggested that the Wnt/β-catenin pathway was enriched in LC according to the GSE101728 and GSE138178 data analysis outcomes, and SESN3 was enriched in this pathway as well. The Wnt/β-catenin pathway, which plays critical roles in embryogenesis, cell proliferation and tumor biology, is commonly deregulated in human cancers [40]. There is no exception for LC, since this pathway is universally activated in LC and is involved in maintenance of cancer initiating cells, tumor progression, metastasis and drug resistance [41]. Our subsequent experiments suggested that the Wnt/β-catenin activity in Hep3B and HLE cells was suppressed after EGR1 knockdown but then recovered after miR-675 upregulation. Further upregulation of SESN3 suppressed the protein levels of β-catenin and c-myc in cells, indicating that EGR1/miR-675/SESN3 axis mediates the Wnt/β-catenin pathway. It can be inferred that downregulation of EGR1 led to a decrease in miR-675 expression and the resultant increase in SESN3 expression, which was responsible for the Wnt/β-catenin inactivation. We surmised that SESN3 possibly suppresses specific key genes of the signaling such as c-myc or to regulate the activity of this signaling pathway, but this needs further validation on a three-dimensional structure. We would like to focus on this issue in our future experiments.

## Conclusion

**Figure 9.**
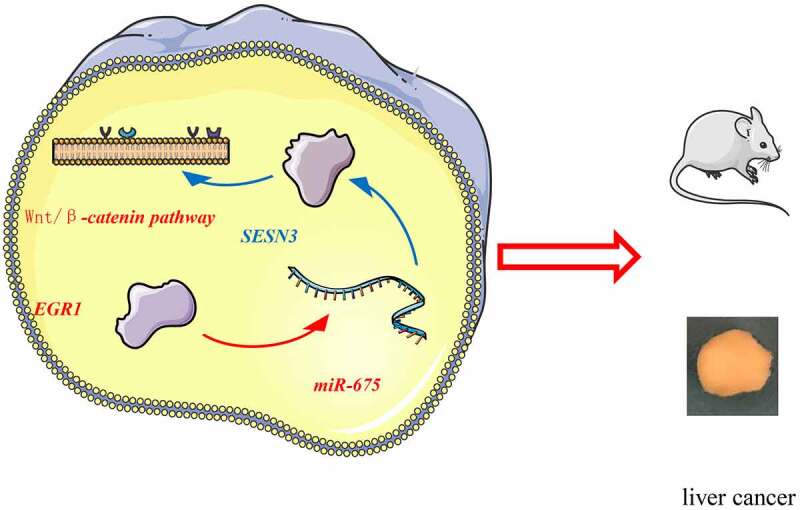
A graphical abstract for the study. In LC, EGR1 is upregulated, which binds to the miR-675 promoter to activate its transcription. miR-675 targets SESN3 mRNA to reduce its expression, which leads to activation of the Wnt/β-catenin and tumor development

To conclude, this study demonstrated that EGR1 binds to the miR-675 promoter to activate its transcription, and miR-675 targets SESN3 mRNA to reduce its expression, which leads to activation of the Wnt/β-catenin (Figure 9). Although the mechanism by which SESN3 inactivates the Wnt/β-catenin remains unclear, this study may provide novel insights into the management of LC that EGR1 and miR-675 may serve as potential targets for LC treatment. We hope more studies will be carried out in the future to examine our findings and to provide more understanding in the molecules involved in LC development.

## Data Availability

All the data generated or analyzed during this study are included in this published article.

## Highlights

- EGR1 is highly expressed in LC samples.
- Downregulation of EGR1 reduces viability of LC cells and tumorigenesis in nude mice.
- EGR1 transcriptionally activates miR-675 to govern SESN3 expression
- miR-675 and SESN3 affects viability and aggressiveness of LC cells.
- The EGR1/miR-675/SESN3 axis mediates the Wnt/β-catenin pathway.
